# Characteristics and factors related to quality of life in Mexican Mestizo patients with celiac disease

**DOI:** 10.1186/s12876-015-0229-y

**Published:** 2015-01-22

**Authors:** Karen Lizzete Ramírez-Cervantes, José María Remes-Troche, María del Pilar Milke-García, Viridiana Romero, Luis F Uscanga

**Affiliations:** 1 Department of Gastroenterology, Instituto Nacional de Ciencias Médicas y Nutrición Salvador Zubirán, Mexico, Mexico; 2 Digestive Physiology and Gastrointestinal Motility Laboratory, Medical and Biologic Research Institute, Universidad Veracruzana, Veracruz, Mexico; 3 National System of Level 1 Researchers, Veracruz, Mexico; 4 Facultad de Medicina, Miguel Alemán Valdés, Veracruz, Mexico; 5 Department of Nutrition Administration, Instituto Nacional de Ciencias Médicas y Nutrición Salvador Zubirán, Mexico, Mexico

**Keywords:** Celiac disease, Quality of life, Hispanic, Mexico

## Abstract

**Background:**

Celiac disease (CD) is a global health problem and its prevalence is underestimated, especially in Latin American populations. Our aim was to evaluate the clinical features, psychological factors, and health-related quality of life (QoL), before and after diagnosis, in a representative sample of adult Mexican Mestizo patients presenting with CD.

**Methods:**

A cross-sectional analysis was conducted on patients seen at two tertiary referral centers in Mexico. QoL before and after CD diagnosis was evaluated using the EuroQoL 5D, the Hospital Anxiety and Depression Scale (HADS), and the disease-specific Celiac Symptom Index (CSI) questionnaires.

**Results:**

We included 80 patients (80% were women, with a mean age of 48.6 ± 14.1 years). The most common symptoms were diarrhea (86%), bloating (77.5%), and abdominal pain (71.3%). Mean symptom duration was 10.33 ± 6.3 years. Fifty-one patients (63.8%) had a previous diagnosis of irritable bowel syndrome (IBS) and 23 (28.8%) had one of functional dyspepsia. Questionnaire respondents rated their health status at 50% before diagnosis (0 = worst imaginable state, 100 = best imaginable state) and there was a significant improvement of 26% after diagnosis. Thirty-nine percent of the patients had a CSI score > 45 and they were the ones that had been previously diagnosed most often with IBS (p = 0.13) or dyspepsia (p = .036).

**Conclusions:**

At the time of diagnosis, Mexican Mestizo patients with CD had poor QoL. Long-standing symptoms and a previous diagnosis of functional disorders were associated with worse QoL. As in other populations, our results support the need for a detailed examination of cost-effective strategies for increasing CD awareness in clinical practice.

## Background

Celiac disease (CD) is a chronic, immune-mediated enteropathy of the small intestine precipitated by exposure to dietary gluten in genetically predisposed individuals [1,2]. Its typical clinical presentation is characterized by signs or symptoms of malabsorption (anemia, abdominal pain, chronic diarrhea, or malnutrition) as a consequence of chronic inflammation and villous atrophy of the small bowel mucosa [1,2]. However, this clinical picture has changed over time and, at present, a considerable number of patients do not have gastrointestinal symptoms, but instead, present with atypical symptoms or no symptoms at all. Because of this heterogeneous clinical picture, a large proportion of patients remain unrecognized. Prompt diagnosis is required in order to prevent chronic complications such as osteoporosis, anemia, malnutrition, and in some cases, malignancies such as intestinal lymphomas. In addition, the symptoms of undiagnosed celiac disease are associated with a prolonged and substantial decrease in quality of life (QoL) that can be similar to or worse than that of subjects with irritable bowel disease (IBS), when compared with healthy controls [3,4]. Previous studies have associated a reduced quality of life in CD patients with abdominal pain, bloating, psychological distress, anxiety, and depression [5-7].

Once diagnosed adherence to treatment involving the lifelong elimination of gluten (gluten-free diet or GFD) results in significant clinical improvement in most patients. However, even though the majority of patients do quite well with a GFD treatment is burdensome in terms of increased cost [8], reduced nutritional value [9], and social constraints [6,10-12].

Recent studies have shown that CD is a global health problem and prevalence is estimated at between 1:250 and 1:67 in some populations [13,14]. Actual prevalence may be underestimated in some areas, such as Latin America or in Hispanic populations [1]. It has recently been reported that seroprevalence for CD using both anti-transglutaminase (tTg-IgA) and anti-endomysial antibodies (EMA) is 0.59% (0.27%-1.29%) in the Mexican Mestizo population [15]. Furthermore, two recent studies have characterized the genetic profile of CD in Mexico [16,17]. Sotelo et al. found HLA DQ2/DQ8 haplotypes in 11 out of 15 children with CD in Northern Mexico [16] and Cerda et al. found a more frequent expression of DQ8 haplotypes in adults with CD [17].

Thus, prevalence and genetic susceptibility in Mexican Mestizo CD subjects is similar to that observed by others, including that of the general U.S. population [1]. However, there is a lack of information related to the characteristics and factors that may have an impact on QoL in Hispanic patients with CD. Our aim was to evaluate the clinical features, psychological factors, and health-related QoL, before and after diagnosis, in a representative sample of adult Mexican Mestizo patients with CD.

## Methods

Between January and August of 2011, patients with CD were recruited at two different tertiary referral centers: 1) consecutive CD patients seen at the *Instituto Nacional de Ciencias Médicas y Nutrición Salvador Zubirán*, and 2) consecutive patients attended to at the *Instituto de Investigaciones Medico Biologicas*, *Universidad Veracruzana*, Veracruz, Mexico. CD diagnosis was established based on the following criteria: 1) compatible clinical characteristics, 2) positive EMA and/or tTg IgA antibodies, and 3) suggestive histological features according to the Marsh Classification.

The Mexican Mestizo is a complex mixture of European (Caucasian) and Native American (Mongoloid) genetics, constituting the core of Mexican and Latin American populations [18]. The Mexican Mestizos in this study were defined as individuals that came from two generations, including their own, of persons born in Mexico, descendants of the original autochthonous inhabitants of the region, as well as of white, mainly Spanish, or black African individuals that came to America in the 16th century [18].

A cross-sectional analysis was carried out using a specific, multiple choice questionnaire for collecting the information in relation to demographics, family history, symptoms experienced before CD diagnosis (diarrhea, bloating, abdominal pain, fatigue, early satiety and/or postprandial fullness [dyspeptic symptoms], weight loss, constipation, headache, skin rashes) and their duration, a prior diagnosis of anemia, osteoporosis, infertility, or recurrent abortion, as well as alternative diagnoses made by attending physicians, especially IBS or dyspepsia, and the number of symptom-related consultations. QoL was evaluated through the EuroQoL 5D (EQ-5D validated Spanish version) [19]. At the time of the study, all the patients had been on a GRD for at least 6 months. The EQ-5D includes both a descriptive system comprised of five health-related domains (mobility, self-care, usual activities, pain/discomfort, and anxiety/depression) divided into three levels of severity (no problems, some problems, severe problems) and a visual analogue scale (VAS) for recording overall health [19]. Patients answered both the descriptive questions and the VAS in relation to two distinct moments in time: “before” and “after” the definitive diagnosis of CD.

The Celiac Symptom Index (CSI), a disease-specific questionnaire [20], was also part of the evaluation. It allows for disease-specific monitoring of symptoms as an independent outcome measure or as part of a surrogate for disease activity in individuals with CD. It is made up of sixteen items, 11 of which evaluate “specific symptoms” and 5 evaluate “general health”. Each question is answered on a Likert scale from 1 to 5. Overall symptom scores were calculated through simple addition, with higher scores denoting more severe symptoms. According to previous studies, scores of 45 or higher are associated with a relatively poor quality of life and worse GFD adherence [20]. Therefore, we analyzed subjects with a CSI > 45.

The Hospital Anxiety and Depression Scale (HADS) was also applied. This scale has been used in Mexico with drug abusers, burn patients, renal insufficiency patients, and obese subjects [18]. According to previous studies, 8 is the best cut-off point for anxiety and 7 for depression. We instructed the patients to answer the HADS questionnaire in relation to their symptoms before CD diagnosis.

The Ethics Committee and the Institutional Review Board at the “*Instituto de Investigaciones Médico Biológicas*”, *Universidad Veracruzana*, Mexico, approved the study. All participants signed statements of informed consent as voluntary participants in the study.

### Statistical analysis

The categorical variables were presented as absolute and relative frequencies, whereas the continuous variables were summarized as means ± SD. The overall prevalence was reported using relative frequencies with the corresponding 95% confidence intervals (based on the binomial distribution). The Student’s t test and Mann–Whitney U test were used for evaluating the continuous data; the chi-square and Fisher exact tests were used for the categorical data. Statistical significance was set at a p <0.05. Correlations between the EQ-D5, VAS, and HADS were calculated with the Spearman’s rho. Data were analyzed using the SPSS software (SPSS 10, Chicago, IL and NCSS-200, Kaysville, UT).

## Results

One hundred and eight subjects were invited to participate in the study, but complete information was provided by 80 subjects (74%). Sixty-four of the respondents were women (80%) and 16 were men (20%), with a mean age of 48.6 ± 14.1 years (range: 23–75 years). The most common symptoms were diarrhea (86%), bloating (77.5%), abdominal pain (71.3%), fatigue (67.5%), dyspeptic symptoms (65%), weight loss (62.5%), constipation (37.5%), headache (36.3%), skin rashes (33.8%), aphthous ulcers (30%), chronic anemia (26.3%), osteoporosis (17.5%), infertility (10%), and recurrent abortion (10%). Seven subjects (9.09%) had a family history of CD (Table 1).

**Table 1:** **Characteristics of CD subjects**

Variable	n = 80
Sex (n,%)	
Men	16 (20%)
Women	64 (80%)
Age (years, mean, SD)	48.6 ± 14.1
Duration of symptoms prior to diagnosis (years, mean, SD)	10.33 ± 6.3
Diarrhea	69 (86%)
Bloating	62 (77.5%)
Abdominal pain	57 (71%)
Fatigue	54 (67.5%)
Dyspeptic symptoms	52 (65%)
Weight loss	50 (62.5%)
Constipation	30 (37.5%)
Headache	29 (36.3%)
Skin rashes	27 (33.8%)
Aphtous ulcers	24 (30%)
Anemia	21 (26.3%)
Osteoporosis	14 (17.5%)
Infertility	8 (10%)
Recurrent abortion	8 (10%)
Family history of CD (n,%)	7 (9%)
Previous diagnosis of IBS	23 (29%)

CD = celiac disease, IBS = irritable bowel syndrome, SD = standard deviation.

Mean symptom duration before diagnosis was 10.33 ± 6.3 years (range: 1–16 years). On average, patients consulted 6.6 physicians prior to correct diagnosis. Correct diagnosis was made by a gastroenterologist in 56 cases, an internist in 10 cases, a nutritionist in 10 cases, and a dermatologist in 4 cases. Ninety percent of the patients (n = 72) stated that information regarding the disease was limited in Mexico and 73% (n = 58) are currently consulting a dietician.

Fifty-one patients (63.8%) had a previous diagnosis of IBS and 23 (28.8%) of functional dyspepsia. The CD subjects with an initial IBS diagnosis were older (43.3 vs. 28.9 years, p = 0.004), went to more consultations (8 vs. 4 visits per year, p = 0.05), and had a longer period of time with symptoms (10.5 vs. 7.25 years, p = 0.06) than those without that initial diagnosis.

**Figure 1:**
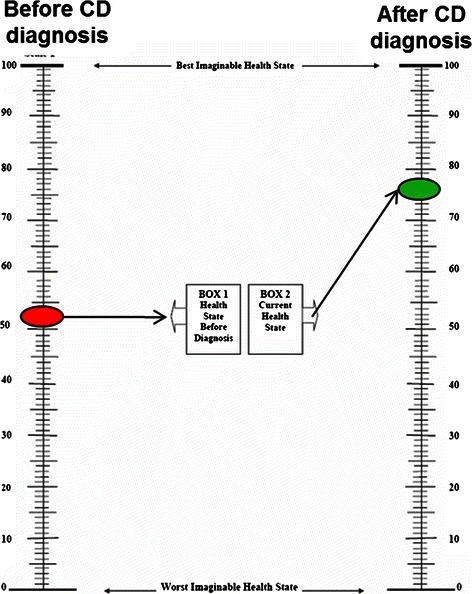
**Visual analogue scale scores before and after celiac disease diagnosis (0 =** **worst imaginable state,** **100 =** **best imaginable state).**

The EQ-5D respondents’ self-reported health status, before and after celiac disease diagnosis, is shown in Table 2. After CD diagnosis, the patients reported a mean improvement in overall health status of 26 points through the VAS (50 vs. 76) (Figure 1).

**Table 2:** **EQ**-**5D respondents**’ **self**-**reported health status before diagnosis of celiac disease (retrospective) and at the present time**: **number and percentage by response level**

EQ-5D question	Level 1		Level 2		Level 3	
	No problems		Some problems		Severe problems	
	n=%		n=%		n=%	
**Mobility**						
Before diagnosis	54	68	23	29	2	3
At present	69	86*	11	14	0	0
**Self-care**						
Before diagnosis	72	90	7	9	1	2
At present	77	96*	3	4	0	0
**Usual activities**						
Before diagnosis	50	62	23	29	7	9
At present	67	84*	11	14	1	2
**Pain**						
Before diagnosis	14	18	42	52	24	30
At present	45	56*	30	37	5	7
**Anxiety/depression**						
Before diagnosis	35	44	31	39	14	17
At present	54	68*	23	30	2	3

*Proportion reporting no problem significantly different at present compared with before diagnosis (McNemar-Bowker Chi-square test, p <0.01).

At the time of the evaluation, 20 patients had a CSI score > 45 (15 women); they were the subjects that had an initial diagnosis of IBS (p = 0.13) or dyspepsia (p = .036), had symptoms for a long period of time (p = 0.04), had more medical consultations (p = 0.024), and displayed a higher prevalence of anxiety and depression (0.003) (Table 3).

**Table 3:** **Factors associated with a celiac score Index higher than 45**

Variable	CSI > 45	CSI < 45	p value
	n = 20	n = 60	
**Sex**			
Men (n,%)	5 (25)	11 (19)	0.23
Women (n,%)	15 (75)	49 (81)	
**Age**			
Mean (SD, years)	43.2 ± 12	40.9 ± 17	0.56
**Duration of symptoms prior to diagnosis**			
Mean (SD, years)	11.8 ± 4	6.8 ± 5.6	0.0001
**Previous IBS diagnosis**			
Yes (n,%)	17 (85)	34 (57)	0.04
No (n,%)	3 (15)	26 (43)	
**Previous dyspepsia diagnosis**			
Yes (n,%)	14 (70)	9 (15)	<0.0001
No (n,%)	6 (30)	51 (85)	
**Pain**			
At time of survey	11 (55)	22 (36)	0.23
**Anxiety or depression**			
At time of survey	14 (70)	18 (30)	0.003

**Figure 2:**
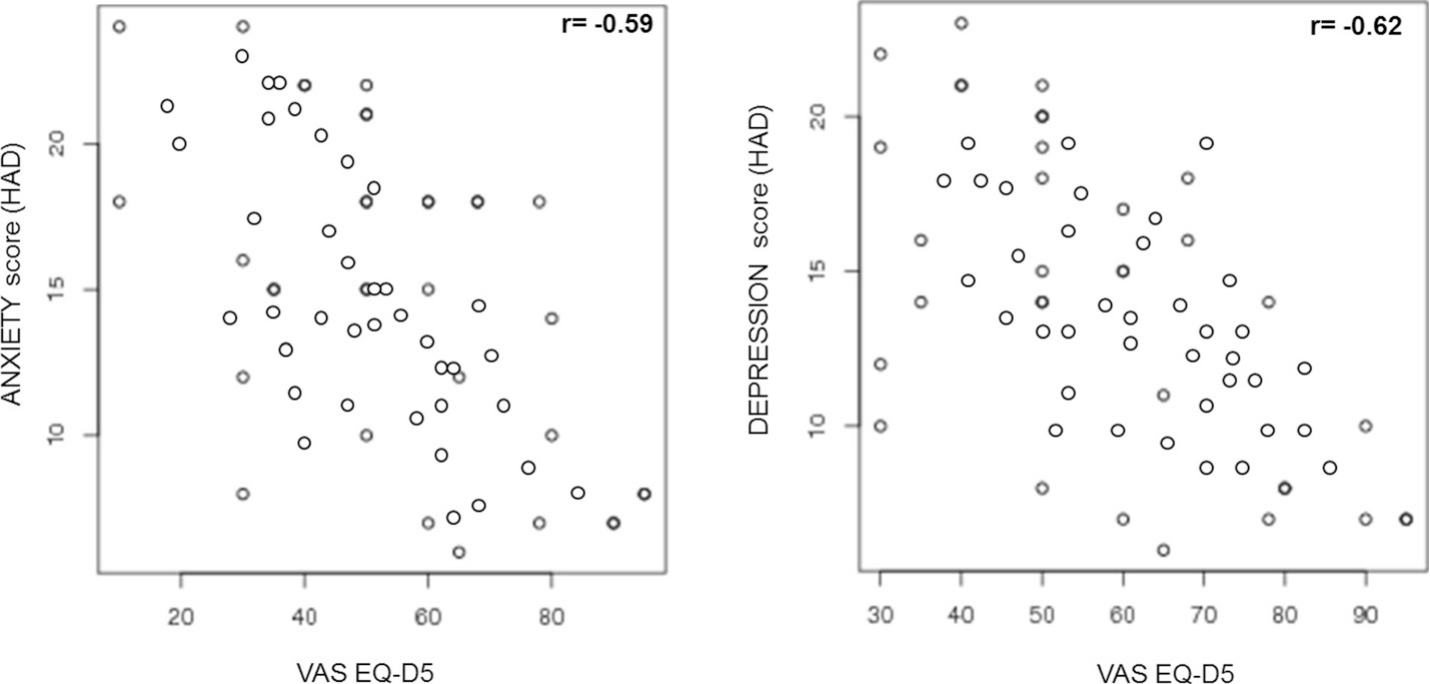
**A significant negative correlation between QoL (** **using the EuroQoL VAS,** **post-** **diagnosis)** **and anxiety and depression** **(r = −** **0.59 and r = −** **0.62)** **was found.**

According to the HADS, 65% (n = 52) presented with anxiety and 60% (n = 48) with depression. There was a negative correlation between QoL (using the VAS, post-diagnosis) and anxiety and depression (r = −0.59 and r = −0.62) (Figure 2).

## Discussion

Although CD was traditionally considered a frequent disease in North America and Europe, recent prevalence studies have indicated that it could be a common health problem worldwide [13,14,19]. To the best of our knowledge, our study is the first characterization of the clinical features, psychological factors, and QoL in adult Mexican Mestizos with CD. In the past, CD was considered an uncommon disease in Mexico, but recent studies have shown that its prevalence in Mexico is at least similar to that of some non-Hispanic countries [1,15].

Like other CD populations, most of our patients had typical symptoms (diarrhea, bloating, abdominal pain, fatigue, dyspeptic symptoms, and weight loss). However, up to 30% had atypical manifestations, such as constipation and dermatologic manifestations. Interestingly, as reported in other countries, Mexican patients with CD may also suffer from other extraintestinal manifestations, such as osteoporosis, infertility, or recurrent abortion [1,2,20]. Thus, even in Mexico, atypical CD is increasing, most likely related to the widespread use of serologic testing and disease awareness in special populations.

As is the case in other ethnic groups, most of our patients are middle-aged women whose diagnosis has been considerably delayed [20]. Our study found a mean symptom duration before diagnosis of 10.32 years; this time lapse is similar to that reported by Green et al. in a large American study [4] and by Gray et al. in a recent British cohort [7]. This delay in CD diagnosis could possibly be explained by the limited availability of serologic tests or by myths and misconceptions regarding CD. For instance, some common CD myths in Mexico are: “The Hispanic population is protected against CD by its dietary staples” or “CD is a disease that only affects Caucasians”, or “there is no known genetic susceptibility related to HLA genes in Hispanics”. In addition, the high rate of misdiagnoses is due to a lack of knowledge among healthcare workers about the disease.

Once they are referred to a gastroenterologist, a substantial number of patients complaining of symptoms suggestive of IBS may actually turn out to have CD [21]. In our study, 64% of the subjects had a previous diagnosis of IBS. Characteristically, these patients were older (p = 0.004), had more consultations (p = 0.05), and had a long history of symptoms (p = 0.06). Similar findings have been reported by other authors. In a large, biopsy-confirmed study that was carried out by means of a postal survey (n = 225), Barrat et al. found that 44% of the CD subjects had a previous IBS diagnosis [22]. A more recent study reported that 16% of individuals with CD had this same prior diagnosis, compared with 4.9% of controls; many of the IBS diagnoses were made within the year before CD diagnosis, but there was a clear excess of IBS diagnoses as much as 10 years earlier [23]. According to our results, an excess of prior IBS diagnoses is related to a diagnostic delay for CDin Mexico; this has also been reported in the UK and other countries. Therefore, healthcare providers should now be aware of this situation.

IBS is not the only functional gastrointestinal disorder that may serve as a reservoir for CD patients. The symptoms of CD can also overlap with those of functional dyspepsia. CD prevalence in dyspepsia has been reported at 1.2-6.2% and may be higher if the entire spectrum of lesions related to gluten sensitivity is taken into account [24]. In our study, 23 (28.8%) of the patients had been diagnosed with functional dyspepsia 3.6 years before being diagnosed with CD.

In regard to QoL determination through the EQ-5D, we found that Mexican CD patients had a poor QoL before CD diagnosis. Several studies have shown that QoL improves once patients are aware of the diagnosis and as soon as they start a GFD [1,25]. For example, one study in the UK concluded that the health-related QoL before CD diagnosis was quantitatively similar to that of stroke patients, and it improved after treatment with a GFD was begun, with scores as good as those of the general population [7]. In our study, there was a significant increase after diagnosis in the percentage of patients that reported having no problems in any of the 5 domains of the EQ-D5. Furthermore, there was a statistically significant increase after diagnosis in the mean value of the pre-diagnosis QoL using the visual scale of the EQ-D5 (from 50 to 76 points). Our results are similar to those reported by Norström et al. in a Swedish cohort of 1,031 CD patients, in which the mean QoL score during the year prior to treatment initiation was low (66 points) and it improved after diagnosis and treatment, reaching 86 points (an increase of 20 points) [3].

In relation to the CSI, 20 of our patients (39.2%) had a score > 45, which has been associated with poor Qol [12]. Previous studies report that women with treated CD experience worse QoL than men. However, in our study, sex was not associated with poor QoL. As has been previously reported [26,27], we found that the delay in CD diagnosis was markedly associated with worse QoL. Another important predictor of reduced QoL was the high prevalence of IBS and dyspepsia symptoms among patients with CD [28,29]. The presence of functional gastrointestinal symptoms in patients with CD is associated with reduced QoL, making those patients more likely to present with anxiety and depression, or vice versa [27,28].

Negative psychological disorders such as anxiety and depression have also been found to be associated with reduced QoL among patients with CD [30]. In a recent meta-analysis, 18 studies on depression and 11 studies on anxiety in adult CD subjects reliably showed that depression was more common and/or more severe in adults with CD than in healthy adults (overall meta-analysis effect size: 0.97) [31]. In a study by Häuser et al. [28], 441 patients with CD and 441 subjects from the general population were assessed by the HADS. The prevalence of anxiety disorder found in the persons with CD (16.8%) was higher than that of the general population (5.7%) (p < 0.001). In our study, according to the HADS, 65% of the CD patients presented with anxiety and 60% with depression at the time of the analysis, and there was a negative correlation with QoL using the EQ-D5 VAS.

Currently, the only treatment for celiac disease is a strict life-long GFD, and despite the fact that this approach has been shown to be beneficial, the diet is troublesome, sometimes expensive, and socially restrictive [25,26]. These negative effects, together with a late diagnosis of the disease, have a negative influence on the QoL of individuals with CD [25]. In relation to our study, we believe that although the QoL score in our patients increased after diagnosis (using the EQ-5D), the prevalence of anxiety and depression remained higher, partially due to the social and cultural issues involved. In addition, the lack of information motivating adherence to a GFD may play a role in a country such as Mexico.

Even though our study presents novel and relevant data on Mexican Mestizo patients with CD, we recognize its limitations. In the past, CD was regarded as an infrequent disease in the Mexican population, and most of the cases were diagnosed at a tertiary referral center such as ours. This could result in selection bias due to the fact that those persons with more severe disease sought medical attention. However, CD awareness is increasing in Mexico and we are now seeing patients with a wider spectrum of CD manifestations, ranging from patients presenting with classic GI symptoms to those with extraintestinal manifestations, such as infertility.

Questionnaires are limiting in general, as there may be bias, particularly when addressing specific aspects. For example, in our study, QoL before and after diagnosis is conducive to recall bias, and the correlation between anxiety, depression, and QoL, before and after diagnosis, could be affected by such bias.

## Conclusions

Nevertheless, our study is the first formal characterization of Mexican CD patients and it showed that long-standing undiagnosed CD and the previous diagnosis of functional disorders were associated with worse QoL in the study population. Our results support the need for a detailed examination of cost-effective strategies for increasing awareness about under-diagnosed CD in clinical practice in Mexico.

## References

[1] Ludvigsson JF, Leffler DA, Bai JC, Biagi F, Fasano A, Green PH, Hadjivassiliou M, Kaukinen K, Kelly CP, Leonard JN, Lundin KE, Murray JA, Sanders DS, Walker MM, Zingone F, Ciacci C (2013). The Oslo definitions for coeliac disease and related terms. Gut.

[2] Barton SH, Murray JA (2008). Celiac disease and autoimmunity in the gut and elsewhere. Gastroenterol Clin N Am.

[3] Norström F, Lindholm L, Sandström O, Nordyke K, Ivarsson A. Delay to celiac disease diagnosis and its implications for health-related quality of life. BMC Gastroenterol. 2011;11:118. 10.1186/1471-230X-11-118 PMC3233515 22060243

[4] Green PHR, Stavropolous SN, Panagi SG, Goldstein SL, Mcmahon DJ, Absan H, Neugut AI (2001). Characteristics of and adult celiac disease in the USA: results of a national survey. Am J Gastroenterol.

[5] Häuser W, Janke KH, Klump B, Gregor M, Hinz A (2010). Anxiety and depression in adult patients with celiac disease on a gluten-free diet. World J Gastroenterol.

[6] Paavola A, Kurppa K, Ukkola A, Collin P, Lähdeaho ML, Huhtala H, Mäki M, Kaukinen K (2012). Gastrointestinal symptoms and quality of life in screen-detected celiac disease. Dig Liver Dis.

[7] Gray AM, Papanicolas N. Impact of symptoms on quality of life before and after diagnosis of celiac disease: results of a UK population survey. BMC Health Serv Res. 2010;10:105. 10.1186/1472-6963-10-105 PMC2907763 20423498

[8] Dorn SD, Hernandez L, Minaya MT, Morris CB, Hu Y, Lewis S, Leserman J, Bangdiwala SI, Green PH, Drossman DA (2010). Psychosocial factors are more important than disease activity in determining gastrointestinal symptoms and health status in adults at a celiac disease referral center. Dig Dis Sci.

[9] Lee AR, Ng DL, Diamond B, Ciaccio EJ, Green PH (2012). Living with coeliac disease: survey results from the USA. J Hum Nutr Diet.

[10] Lee AR, Ng DL, Zivin J, Green PH (2007). Economic burden of a gluten-free diet. J Hum Nutr Diet.

[11] Thompson T, Dennis M, Higgins LA, Lee AR, Sharrett MK (2005). Gluten-free diet survey: are Americans with coeliac disease consuming recommended amounts of fibre, iron, calcium and grain foods?. J Hum Nutr Diet.

[12] Leffler DA, Dennis M, Edwards George J, Jamma S, Cook EF, Schuppan D, Kelly CP (2009). A validated disease-specific symptom index for adults with celiac disease. Clin Gastroenterol Hepatol.

[13] Fasano A, Berti I, Gerarduzzi T, Not T, Colletti RB, Drago S, Elitsur Y, Green PH, Guandalini S, Hill ID, Pietzak M, Ventura A, Thorpe M, Kryszak D, Fornaroli F, Wasserman SS, Murray JA, Horvath K (2003). Prevalence of celiac disease in at-risk and not-at-risk groups in the United States: a large multicenter study. Arch Intern Med.

[14] Rubio-Tapia A, Ludvigsson JF, Brantner TL, Murray JA, Everhart JE (2012). The prevalence of celiac disease in the United States. Am J Gastroenterol.

[15] Remes-Troche JM, Nuñez-Alvares C, Uscanga-Dominguez LF (2013). Celiac disease in Mexican population: an update. Am J Gastroenterol.

[16] Sotelo Cruz N, Calderón De La Barca AM, Hurtado Valenzuela JG (2013). Celiac disease in children from the northwest of Mexico: clinical characteristics of 24 cases. Rev Gastroenterol Mex.

[17] Cerda-Contreras E, Duarte-Rojo A, Granados J, Vargas F, Uscanga-Dominguez LF (2008). Frecuencia de antígenos de histocompatibilidad DQ2 DQ8 en sujetos con diarrea crónica y Enfermedad Celiaca (EC). Rev Gastroenterol Mex.

[18] Yamamoto-Furusho JK, Uscanga-Domínguez L, Lopez-Martinez A, Granados J (2006). Association of the HLA-DRB1*0701 allele with perinuclear anti-neutrophil cytoplasmatic antibodies in Mexican patients with severe ulcerative colitis. World J Gastroenterol.

[19] Brooks R (1996). EuroQol: the current state of play. Health Policy.

[20] López-Alvarenga JC, Vázquez-Velázquez V, Arcila-Martínez D, Sierra-Ovando AE, González-Barranco J, Salín-Pascual RJ (2002). Accuracy and diagnostic utility of the Hospital Anxiety and Depression Scale (HAD) in a sample of obese Mexican patients. Rev Invest Clin.

[21] Kang JY, Kang AH, Green A, Gwee KA, Ho KY (2013). Systematic review: worldwide variation in the frequency of coeliac disease and changes over time. Aliment Pharmacol Ther.

[22] Ciacci C, Cirillo M, Sollazzo R, Savino G, Sabbatini F, Mazzacca G (1995). Gender and clinical presentation in adult celiac disease. Scand J Gastroenterol.

[23] Sainsbury A, Sanders DS, Ford AC (2013). Prevalence of irritable bowel syndrome- type symptoms in patients with celiac disease: a meta-analysis. Clin Gastroenterol Hepatol.

[24] Barratt SM, Leeds JS, Robinson K, Lobo AJ, McAlindon ME, Sanders DS (2011). Prodromal irritable bowel syndrome may be responsible for delays in diagnosis in patients presenting with unrecognized Crohn’s disease and celiac disease, but not ulcerative colitis. Dig Dis Sci.

[25] Card TR, Siffledeen J, West J, Fleming KM (2013). An excess of prior irritable bowel syndrome diagnoses or treatments in Celiac disease: evidence of diagnostic delay. Scand J Gastroenterol.

[26] Santolaria Piedrafita S, Fernández BF (2012). Gluten-sensitive enteropathy and functional dyspepsia. Gastroenterol Hepatol.

[27] Murray JA, Watson T, Clearman B, Mitros F (2004). Effect of a gluten free diet on gastrointestinal symptoms. Am J Clin Nutri.

[28] Casellas F, Rodrigo L, Vivancos JL, Riestra S, Pantiga C, Baudet JS, Junquera F, Diví VP, Abadia C, Papo M, Gelabert J, Malagelada JR (2008). Factors that impact health-related quality of life in adults with celiac disease: a multicenter study. World J Gastroenterol.

[29] Kurien M, Barrat SM, Sanders DS (2011). Functional gastrointestinal disorders in adults-negative impact on quality of life. Aliment Pharmacol Ther.

[30] Barratt SM, Leeds JS, Robinson K, Shah PJ, Lobo AJ, McAlindon ME, Sanders DS (2011). Reflux and irritable bowel syndrome are negative predictors of quality of life in celiac disease and inflammatory bowel disease. Eur J Gastroenterol Hepatol.

[31] Smith DF, Gerdes LU (2012). Meta-analysis on anxiety and depression in adult celiac disease. Acta Psychiatr Scand.

